# Eyelid Contact Dermatitis: 25-Year Single-Center Retrospective Study

**DOI:** 10.3390/jcm14030823

**Published:** 2025-01-27

**Authors:** Giovanni Rubegni, Tommaso Padula, Laura Calabrese, Martina D’Onghia, Linda Tognetti, Elisa Cinotti, Laura Lazzeri, Gabriele Ermini, Alessandra Cartocci, Gian Marco Tosi

**Affiliations:** 1 Ophthalmology Unit, Department of Medicine, Surgery and Neurosciences, University of Siena, 53100 Siena, Italy; tommasopadula0498@gmail.com (T.P.); gianmarco.tosi@unisi.it (G.M.T.); 2 Department of Medical, Surgical and Neurological Sciences, Dermatology Section, University of Siena, 53100 Siena, Italymartina.donghia@gmail.com (M.D.); linda.tognetti@dbm.unisi.it (L.T.); elisa.cinotti@unisi.it (E.C.); lazzeri.laura@virgilio.it (L.L.); alessandra.cartocci@dbm.unisi.it (A.C.); 3 F.I.R.M.A. S.p.A., Allergologia e Immunologia Clinica, 50100 Firenze, Italy; gabryermini@gmail.com

**Keywords:** eyelid skin lesions, allergy, allergic contact dermatitis, ophthalmoplasty, inflammatory skin diseases, hapten

## Abstract

**Background/Objectives**: Eyelid dermatitis is an inflammatory disease affecting the palpebral skin characterized by itching, edema, and scaling of the periorbital area. This entity can be a manifestation of various underlying dermatological diseases, but allergic contact dermatitis (ACD) is the predominant etiology of eyelid dermatitis among patients, being diagnosed in 43.4% of cases. The thin and highly permeable nature of eyelid skin increases its susceptibility to allergens, making it a distinct clinical entity. This study aimed to identify the primary haptens associated with eyelid ACD and compare these findings with the allergens implicated in non-eyelid ACD over a 25-year period in a large cohort of patients. **Methods**: We conducted a monocentric, retrospective study on a dataset of 7955 patients patch-tested for ACD at the Outpatient Allergy Dermatology Clinic of the Azienda Ospedaliera Universitaria Senese (AOUS) from 1997 to 2021. Eyelid ACD cases were identified based on clinical features and positive patch test results. Data on demographics, occupation, and personal history of atopy were collected. The statistical analyses assessed the associations between allergens and eyelid ACD. The trends in the sensitization rates for the most prevalent allergens were also evaluated. **Results**: Eyelid ACD was identified in 4.6% of the study population, predominantly affecting women (88.6%). Patients with eyelid ACD were more likely to exhibit single-hapten positivity (54.6%) and an atopic phenotype (52.3%) compared to non-eyelid ACD cases. Nickel sulfate (54%), cobalt chloride (13.4%), and thimerosal (12.6%) were the most common allergens associated with eyelid ACD. While thimerosal sensitization decreased significantly following its removal from topical products, nickel sensitization increased, likely due to exposure from electronic devices and hand–eye contact. **Conclusions**: The haptens identified in eyelid ACD largely overlap with those found in other body regions, including metals, fragrances, and preservatives. However, the unique characteristics of eyelid skin and hand–eye contact patterns play a significant role in sensitization. This study highlights the need for further investigation into the pathophysiology of eyelid allergic contact dermatitis, with particular emphasis on elucidating the mechanisms of hapten sensitization. Such insights could contribute to the development of effective strategies aimed at reducing allergen exposure.

## 1. Introduction

Eyelid dermatitis is an inflammatory condition affecting the palpebral skin, characterized by itching, edema, and scaling of the periorbital area [1]. This condition can manifest as a result of various underlying dermatological diseases, including atopic dermatitis (AD,) allergic contact dermatitis (ACD), irritant contact dermatitis (ICD), seborrheic dermatitis, rosacea, infections, and dermatomyositis.

AD is a chronic, relapsing inflammatory skin disease, commonly observed in children, and results from a combination of genetic predisposition, skin barrier dysfunction, immune dysregulation, and environmental factors. ACD, on the other hand, is an inflammatory skin disorder caused by a type IV hypersensitivity reaction triggered by exposure to a specific substance (hapten). In ICD, skin inflammation arises from direct physical or chemical damage to the epidermis [2].

According to recent studies, ACD represents the predominant cause of eyelid dermatitis, accounting for 43.4% of cases [3]. The histological characteristics of eyelid skin contribute to its susceptibility to ACD, as it possesses a highly hydrated stratum corneum and is the thinnest skin on the body (0.55 mm). These features facilitate the penetration of contact and airborne allergens, rendering the eyelids more vulnerable than other anatomical sites [4].

Although it is non-life-threatening and confined to a small body surface area, periorbital ACD significantly impairs quality of life due to its chronic and recurrent nature [5]. This condition not only affects the aesthetic well-being of patients but also compromises vision, often due to palpebral edema and alterations in tear dynamics [6,7].

Patch testing remains a fundamental diagnostic tool for the early detection and elimination of the causative allergens in allergic eyelid dermatitis, which is crucial for preventing chronicity [8].

The aim of this study was to identify the primary haptens responsible for allergic contact dermatitis (ACD) and examine the changes in their prevalence over a 25-year period. Additionally, it sought to compare the clinical characteristics of eyelid ACD with those of non-eyelid ACD in a large cohort of patients who underwent patch testing at the Outpatient Allergy Dermatology Clinic of the Azienda Ospedaliera Universitaria Senese (AOUS) between 1997 and 2021.

## 2. Materials and Methods

### 2.1. The Case Study

This retrospective, monocentric study was approved by the local Ethics Committee of the University Hospital of Siena (Study Protocol No. 16801) and conducted in accordance with the principles outlined in the Declaration of Helsinki. All of the data were de-identified prior to their use to ensure patient confidentiality.

This study included all patients with a confirmed diagnosis of allergic contact dermatitis (ACD), established based on clinical characteristics and a positive patch test, who were evaluated between 1 January 1997 and 31 December 2021. Eyelid ACD was clinically defined according to the presence of scaling, erythema, and irritation around the eyes, with a positive patch test to at least one allergen. Cases of non-eyelid dermatitis were further classified as diffuse when the inflammatory process, characterized by scaling and irritation, affected more than four body sites and as localized when four or fewer body sites were involved. In localized cases, the specific site of involvement was identified among the following: the trunk, perianal area, breasts, limbs, axillae, oral cavity, feet, neck, ears, scalp, face (excluding the eyelid region), male genital area, hands, eyelids, nails, and vulva/vagina.

All of the patients completed a structured questionnaire collecting information on their occupation, age, sex, symptoms, and personal history of atopy. The occupational categories included healthcare workers, construction workers, metal workers, office workers, farm laborers, textile workers, chemical workers, food industry workers, housewives, and transport workers. The questionnaire also recorded their history of atopic conditions such as atopic dermatitis, allergic rhinitis, and bronchial asthma.

### 2.2. The ACD Test

Most of the individuals underwent patch testing using the standard GIRDCA (Gruppo Italiano Ricerca Dermatiti da Contatto e Ambientali) and SIDAPA (Italian Society of Occupational and Environmental Allergologic Dermatology) baseline series applied over the past 25 years. Among the haptens included in these series, only those that were consistently tested throughout the entire 25-year period were considered in this study. Specifically, the allergens analyzed were perfume mix II (14%), thiuram mix (1%), potassium dichromate (0.5%), Peru balsam (25%), p-Phenylenediamine (1%), colophony (20%), neomycin sulfate (20%), cobalt (II) chloride hexahydrate (1%), formaldehyde (2% H_2_O), p-tert-butylphenol-formaldehyde resin, nickel (II) sulfate hexahydrate (5%), Carba mix (3%), propolis (20%), disperse blue 124, thimerosal, Kathon CG, ammoniated mercury chloride, benzalkonium chloride, benzocaine (5%), and paraben mix (16%). Rare haptens were defined as those eliciting a positive reaction in fewer than 0.5% of the individuals tested.

Prior to testing, all patients were thoroughly informed about the necessary precautions to be followed, including the avoidance of showers, hot baths, exercise, and any activities that could induce sweating and lead to the detachment of the patch test apparatus. Additionally, to prevent false negative reactions, the use of both topical and systemic corticosteroids and antihistamines was strictly prohibited in the two weeks preceding the test.

Patch tests were applied to the upper back using Haye’s test chambers (Haye’s Service B.V., Ampfield, Great Britain) on Soffix tape (PIKDARE Spa, Como, Italy). The allergens, provided by FIRMADiagent, remained occluded for 48 h, and the patch test readings were conducted at 48 h (D2) and 72 h (D3), in accordance with the Italian Guidelines on Patch Testing [8].

### 2.3. Statistical Analysis

Descriptive statistics were calculated, and quantitative variables were summarized as the mean and standard deviation or as the median and interquartile range. Qualitative variables were reported as absolute frequencies and percentages. The Chi-squared test was estimated to evaluate the association between the agents and eyelid ACD. A *p*-value < 0.05 was considered statistically significant. All of the analyses were carried out using R version 4.3.1.

## 3. Results

### 3.1. Sample Characteristics

During the 25-year time window, 7955 patients with suspected ACD were enrolled. A total of 292 out of 7955 had a positive reaction to any agent, while 350 of the remaining 7667 (4.6%) patients had eyelid ACD. The most prevalent patterns were diffuse ACD (52.2%) and hand ACD (22.8%) (see Table 1). The sample was constituted of 23.2% male patients, and the mean age was 42.65 ± 16.91. Office workers and housewives/retirees were the work categories with the highest prevalence of eyelid and non-eyelid ACD (Table 1).

### 3.2. Comparison of Eyelid ACD and Non-Eyelid ACD

The average age was 42.66 ± 16.98 for eyelid ACD and 42.30 ± 15.47 for non-eyelid ACD.

As shown in Table 2, the predominance of the female gender in the eyelid ACD group (88.6%) was statistically significant compared to that in non-eyelid ACD (76.2%) (*p* < 0.001).

The occupations most frequently associated with eyelid ACD were office worker/teacher (48.9%) and housewife/retiree (17.7%). Patients with eyelid ACD more frequently worked in an office than patients with non-eyelid ACD (48.9 vs. 30.8; *p* < 0.001).

The patients with eyelid ACD were more frequently associated with single positivity (54.6% vs. 46.6%, *p* = 0.009).

At the initial visit, the eyelid ACD patients more frequently had an atopic phenotype than those with non-eyelid ACD (52.3% vs. 41.0%, *p* < 0.001).

Among the patients with eyelid ACD, 54% tested positive to a single hapten, compared to 45% of those with ACD affecting other body sites. A total of 23.4% of eyelid ACD cases were positive for two haptens and 22.0% for three or more haptens, compared to rates of 25.1% and 28.2%, respectively, in the non-eyelid forms.

Comparing the prevalence of positive haptens between eyelid ACD and non-eyelid ACD, it was possible to observe a similar prevalence of nickel sulfate (54.0% vs. 53.3%, *p* = 0.837), cobalt chloride (13.4% vs. 16.7%, *p* = 0.124), thimerosal (12.6% vs. 10.3%, *p* = 0.206), Kathon CG (7.1% vs. 7.7%, *p* = 0.795), perfume mix (6.9% vs. 9.9%, *p* = 0.072), disperse blue (4.9% vs. 3.4%, *p* = 0.177), balsam of Perù (4.6% vs. 6.8%, *p* = 0.129), and Carba mix (4.6% vs. 6.6%, *p* = 0.166). Phenylenediamine was significantly more prevalent in the non-eyelid ACD group (Phenylenediamine: 7.4% vs. 3.4%, *p* = 0.007), as were potassium dichromate (10.1% vs. 4.9% *p* = 0.002) and Thiuram mix 1% (3.5% vs. 1.1%, *p* = 0.028); however, benzalkonium chloride was more prevalent in the eyelid ACD group (3.1% vs. 0.4%, *p* < 0.001) (see Table 2).

**Figure 1:**
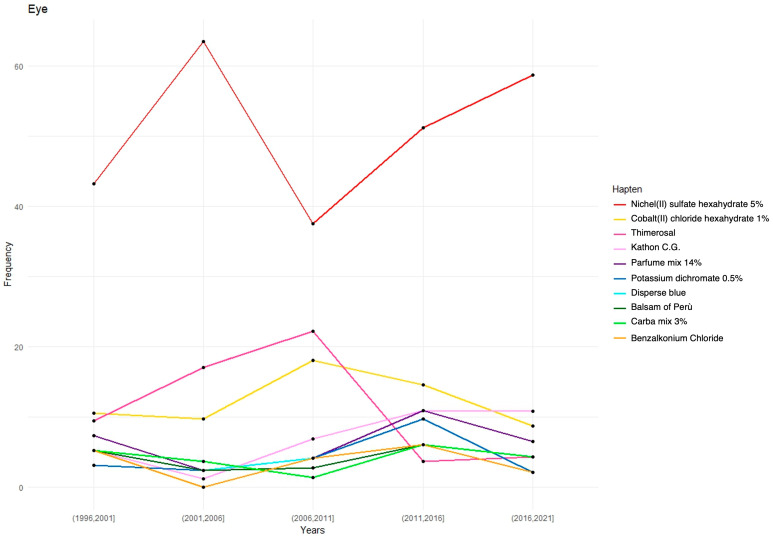
Hapten positivity prevalence over the years.

The prevalence trends for the 10 most frequently positive allergens in the eyelid ACD patients were assessed over the past 25 years (Figure 1).

## 4. Discussion

Allergic contact dermatitis (ACD) is an immune-mediated skin condition characterized by an erythematous and pruritic rash triggered by exposure to specific allergens [2]. While ACD can affect any part of the body, involvement of the eyelids and periorbital skin presents unique clinical characteristics and additional challenges for affected patients [9].

In our study, 4.72% of the patch-tested patients were diagnosed with allergic eyelid dermatitis, of whom approximately 86.4% (326/377) exhibited exclusive eyelid involvement. Compared to recent findings in the literature, this prevalence was lower than the previously reported rates of 7.2% [10], 8.6% [11], and 12.9% [1]. Variations in these prevalence rates may be attributed to differences in sensitization levels across countries and the specialization of certain centers, which may evaluate a more selective cohort of patients at the end of the referral process [1,11,12,13,14].

Our study found that the majority of the patients with allergic eyelid dermatitis were women (88.6% vs. 11.4%), a result consistent with the previous literature that has attributed a higher prevalence of eyelid dermatitis to female patients. This distribution has been hypothesized to result from the increased application of cosmetics and topical products to the face, particularly the periorbital area [3,11,14,15,16,17,18,19]. The mean age in our study was 42.74 ± 15.41 years, and no statistically significant difference was observed in the age at presentation between the patients with and without eyelid dermatitis (*p* = 0.941), a finding consistent with the observations of Kathani et al. [11].

Regarding the body sites associated with eyelid dermatitis, the anatomical areas most affected in patients without eyelid involvement were all significantly less prevalent in the group with eyelid dermatitis (e.g., the limbs, feet, face, and hands). This phenomenon may be explained by the particular vulnerability of the eyelid skin to hapten penetration, which can elicit an immune response even at lower hapten concentrations that may not induce a reaction in other areas of the body [4].

Our findings indicate that patients with eyelid ACD were more frequently atopic than those without eyelid involvement (52.3% vs. 41.0%, *p* < 0.001). Atopic individuals are at a higher risk of developing contact dermatitis due to their unique skin characteristics, including an impaired barrier function, which results in an approximately two-fold higher absorption of irritants and allergenic substances compared to that in non-atopic individuals [20,21,22].

Nickel, often in combination with cobalt chloride cross-sensitization, was the most common allergen among the patients with eyelid dermatitis (50.1%); however, no significant difference was observed compared to patients without eyelid dermatitis (51.4%). Nickel sensitization can result from the direct application of nickel-containing eye products (e.g., mascara, eyeshadow, eyeliners) or contact with metal accessories, such as spectacle frames and eyelash curlers [23,24,25]. Given that most cosmetic products no longer list nickel as an ingredient, exposure is thought to occur primarily through accidental transfer, particularly via hand-to-eye contact [26,27]. It is likely that inadvertent transfer accounts for the majority of nickel-induced periorbital dermatitis cases, as previously reported in individuals with periorbital and eyelid dermatitis [1,28].

Despite a decline in the use of nickel-containing cosmetic products in recent years, our study indicates that nickel sensitization has increased over the past two decades, rising from a 42% positivity rate in 1996–2001 to 59% in 2016–2021. The widespread presence of nickel-containing items (e.g., keys, belts, rings, pens, lighters, zippers, household goods, and electronic devices), in combination with frequent hand-to-eye contact, may explain the rising sensitization rates observed in our study population [27,29].

Similar to nickel, thimerosal yielded a high rate of positive patch test results (11.9%). Previously used as a preservative in cosmetics (e.g., eyeshadows), eye drops, and topical medications, as well as in contact lens solutions [30], thimerosal was removed from topical products after 2003 when the U.S. Subcommittee on Human Rights and Wellness concluded that “…thimerosal is not safe for topical use due to its potential for cell damage when applied to broken skin and its allergenic potential. It is not effective as a topical antimicrobial because its bacteriostatic action can be reversed” [31].

Our study demonstrated a marked decline in thimerosal patch test positivity between 2006 and 2021, likely reflecting the reduction in its use. In contrast to nickel, the elimination of thimerosal from cosmetic products and ophthalmic medications has led to a significant reduction in positive patch test responses among patients with periorbital dermatitis. As previously discussed, this difference is likely attributable to the widespread presence of nickel in common consumer products, whereas thimerosal, solely used as a preservative, was deemed unnecessary in medical and non-medical formulations.

The preservative Kathon CG was also identified as a common sensitizer in our study (6.6%). Since the 1970s, it has been used as a preservative in agricultural and industrial cleaning products (in a fixed 3:1 combination). In 2000, it began to be marketed separately for industrial use in products such as paints and adhesives, and since 2005, it has also been incorporated into cosmetics [32]. Our study revealed a sharp increase in the sensitization rates from 1% in 2001–2006 to 11% in 2016–2021, coinciding precisely with the commercial expansion of cosmetic products containing Kathon CG. These findings are consistent with those of Burnett et al., who documented a significant rise in Kathon CG sensitization over the past two decades [33,34].

The fragrance mix and the cross-reactive balsam of Peru were also among the most frequently identified allergens in our study. These allergens are commonly found in cosmetic products and fragrances [35]. The sensitization rates were 6.4% for the fragrance mix and 4.2% for balsam of Peru, aligning with previous research that has reported positive reaction rates ranging from 8% to 20% for the fragrance mix and 3.3% to 8% for balsam of Peru [1,13].

Unlike thimerosal, these allergens have not been subject to regulatory restrictions in the cosmetics market. Our findings confirm a consistent rate of positive reactions among individuals with eyelid dermatitis from 1996 to 2021. Similarly, the positivity rates for disperse blue, potassium dichromate, and Carba mix have remained stable in recent years.

The only allergen found to be more prevalent in patients with eyelid involvement than in those with dermatitis affecting other body sites was benzalkonium chloride (BAK) (2.9% vs. 0.4%). Currently, BAK is used in approximately 70% of ophthalmic formulations [36]. Despite its widespread use, the rate of sensitization to this preservative remains low and is significantly lower than that for another common eye drop preservative, thimerosal (11.9% vs. 2.9%). BAK reactions tend to be irritant-based, whereas thimerosal reactions are predominantly allergic [30]. Although BAK-free eye drops have become increasingly available in recent years, this preservative remains widely used, and its positivity rate has remained low and stable from 1996 to 2021.

Several limitations of this study should be acknowledged. A key limitation is the retrospective and long-term nature of this study, during which different specialists, working in various settings over time, conducted the patch testing despite standardized clinical protocols. Another important limitation is that patch testing was performed on the upper back, where differences in allergen penetration and skin susceptibility compared to those in the periorbital region may have led to weaker allergens that affect the eyelids not being identified. Lastly, our study population consisted of patients referred for patch testing by dermatologists, which may limit the generalizability of our findings to the broader population.

## 5. Conclusions

In conclusion, most of the allergens seen in eyelid allergic contact dermatitis were comparable to those discovered in other body regions, such as metals (nickel, cobalt), fragrances (fragrance mix and balsam of Perù), and preservatives (thimerosal, BAK). Patch test positivity to ophthalmic allergens is less common than that to environmental allergens, most likely due to hand-to-eye contact sensitization and the increased use of preservative-free ophthalmic drops. The progression of the most frequent types of hapten positivity over time showed that only the removal of thimerosal was actually effective in reducing sensitivity, while nickel increased in its positivity rate despite its removal from cosmetic products over the last 10 years. We hypothesized its presence in electronic devices, which are being used with increasing frequency, and hand–eye contact as the main mechanism of sensitization, but further studies will be necessary to explain this trend.

While diagnosis and management of eyelid dermatitis can be complex, a detailed patient history combined with appropriate patch testing can ensure an accurate diagnosis and effective management.

## Figures and Tables

**Table 1:** Comparison of eyelid ACD and non-eyelid ACD:.

Variables	Non-Eyelid ACD N = 7313	Eyelid ACD N = 350	*p*
Male	1737 (23.8%)	40 (11.4%)	<0.001
Age	42.66 ± 16.98	42.30 ± 15.47	0.697
Diffuse body site	4000 (52.2%)	/	
Specific body site	3667 (47.9%)	/	
Trunk	89 (1.2%)	0 (0.0%)	0.067
Perianal area	43 (0.6%)	3 (0.9%)	0.777
Breasts	23 (0.3%)	0 (0.0%)	0.582
Limbs	414 (5.4%)	2 (0.6%)	<0.001
Axillae	37 (0.5%)	1 (0.3%)	0.854
Oral cavity	135 (1.8%)	6 (1.7%)	1.0
Foot	338 (4.4%)	0 (0.0%)	<0.001
Neck	124 (1.7%)	7 (2.0%)	0.827
Ears	42 (0.6%)	3 (0.9%)	0.750
Scalp	126 (1.7%)	2 (0.6%)	0.153
Face	650 (8.5%)	4 (1.1%)	<0.001
Male genital area	18 (0.3%)	2 (0.6%)	0.529
Hands	1729 (23.6%)	21 (6.0%)	<0.001
Nails	1 (0.0%)	0 (0.0%)	1.0
Vulva/Vagina	46 (0.6%)	0 (0.0%)	0.257
Work			
Chemical worker	395 (5.4%)	15 (4.3%)	0.433
Construction worker	156 (2.1%)	1 (0.3%)	0.029
Farm laborer	129 (1.8%)	5 (1.4%)	0.796
Food worker	88 (1.2%)	4 (1.1%)	1.0
Health worker	278 (3.8%)	9 (2.6%)	0.298
Housewife/retiree	1644 (22.4%)	63 (18.0%)	0.057
Metal worker	160 (2.2%)	4 (1.1%)	0.258
Office worker	2237 (30.6%)	189 (54.0%)	<0.001
Textile worker	152 (2.1%)	6 (1.7%)	0.783
Transport worker	168 (2.3%)	6 (1.7%)	0.595
Other	1894 (25.9%)	64 (18.3%)	<0.001

**Table 2:** Comparison of eyelid ACD and non-eyelid ACD: atopy and haptens.

Variables	Non-Eyelid ACD N = 7313	Eyelid ACD N = 350	*p*
Atopy	2634 (41.4%)	175 (53.8%)	<0.001
Num. hapten positivity (%)num_all (%)			0.009
1	3411 (46.6%)	191 (54.6%)	
2	1839 (25.1%)	82 (23.4%)	
≥3	2063 (28.2%)	77 (22.0%)	
Rare allergen haptens	86 (1.2%)	3 (0.9%)	0.773
Perfume mix II 14%	726 (9.9%)	24 (6.9%)	0.072
Thiuram mix 1%	253 (3.5%)	4 (1.1%)	0.028
Potassium dichromate 0.5%	735 (10.1%)	17 (4.9%)	0.002
Peru balsam 25%	497 (6.8%)	16 (4.6%)	0.129
p-Phenylenediamine 1%	539 (7.4%)	12 (3.4%)	0.007
Colophony 20%	178 (2.4%)	6 (1.7%)	0.496
Neomycin sulfate 20%	186 (2.5%)	10 (2.9%)	0.849
Cobalt (II) chloride hexahydrate 1%	1222 (16.7%)	47 (13.4%)	0.124
Formaldehyde 2% H_2_O	272 (3.7%)	6 (1.7%)	0.070
p-tert-Butylphenol-formaldehyde resin	167 (2.3%)	7 (2.0%)	0.870
Nickel (II) sulfate hexahydrate 5%	3897 (53.3%)	189 (54.0%)	0.837
Carba mix 3%	482 (6.6%)	16 (4.6%)	0.166
Propolis 20%	325 (4.4%)	14 (4.0%)	0.794
Disperse blue 124	246 (3.4%)	17 (4.9%)	0.177
Thimerosal	754 (10.3%)	44 (12.6%)	0.206
Kathon CG	561 (7.7%)	25 (7.1%)	0.795
Ammoniated mercury chloride	139 (1.9%)	7 (2.0%)	1.000
Benzalkonium chloride	31 (0.4%)	11 (3.1%)	<0.001
Benzocaine 5%	80 (1.1%)	2 (0.6%)	0.508
Paraben mix 16%	87 (1.2%)	2 (0.6%)	0.424

## Data Availability

The data are available upon request.
